# Mozambique Field Epidemiology and Laboratory Training Program as responders workforce during Idai and Kenneth cyclones: a commentary

**DOI:** 10.11604/pamj.2020.36.264.21087

**Published:** 2020-08-11

**Authors:** Cynthia Semá Baltazar, Erika Valeska Rossetto

**Affiliations:** 1 Instituto Nacional de Saúde, Maputo, Mozambique,; 2 MassGenics, Assigned to Mozambique Centers for Disease Control Prevention, Maputo, Mozambique

**Keywords:** Epidemiology, emergency responders, public health surveillance, health workforce, cyclones, floods, Mozambique

## Abstract

The ability to rapidly and effectively respond to public health emergencies, including outbreak investigations and natural disasters, is critical in a strengthened health system. In March and April 2019, the impact of tropical cyclones Idai and Kenneth in Southern Africa and subsequent flooding resulted in devastating consequences to the Mozambique health care system. In this article, we highlight the role of Mozambique’s Field Epidemiology and Laboratory Training Program (FELTP) graduates as first responders during one of the most significant natural disasters on the African continent. The FELTP graduates played a key role in conducting risk assessments, active epidemiological surveillance for priority communicable diseases, and outbreak investigations and supporting the laboratory diagnosis system. The cyclone emergencies in Mozambique revealed the vulnerability of the health system. It is vital to continue the investment in increasing epidemiological capacity of health human resources, staff to adequately prepare for and respond to public health emergencies to mitigate the negative health impacts associated with those events.

## Commentary

Mozambique is ranked as the third highest “at-risk” African country most vulnerable to weather-related events such as cyclones, droughts, floods, and the resulting epidemics that often follow natural disasters [1]. On the 14^th^ of March 2019, Tropical Cyclone Idai, made landfall near Beira City, Mozambique’s fourth largest city with an estimated population of 500,000, causing catastrophic flooding and resulting in one of the worst humanitarian crises in Mozambique in recent history. Damage from the cyclone severely disrupted water and food supplies, sanitation, electricity, transportation, shelter, communications, security, medical care, and mosquito control.” The cyclone affected 1.85 million people and brought destruction and damage to five of eleven provinces in central Mozambique including Sofala, Manica, Tete, Zambézia and Inhambane provinces. More than 400,000 people were displaced with around 161,000 people sheltered in 164 temporary accommodation centers. Over 600 people were confirmed dead and more than 1,500 people injured. The Mozambique Government declared a state of emergency, and the World Health Organization (WHO) declared the humanitarian situation in Mozambique as a Grade 3 emergency on March 25^th^, 2019 [2].

On 25^th^ April, six weeks after Cyclone Idai, the Cyclone Kenneth, also category 3, reached the northern coast of Mozambique, affecting Cabo Delgado Province [2]. It was estimated that Cyclone Kenneth affected 254,750 people, 45 people died, and more than 45,00 houses were either completely or partially destroyed. Approximately 18,000 people were displaced and were sheltering in an accommodation center [3]. The health care system was seriously damaged by these cyclones with 112 health facilities damaged or destroyed, further reducing access to health services for communities living in remote and hard-to-reach areas. In view of the mass flooding brought on by both cyclones, as well as structural damage to the water and sanitation system cholera cases were expected. On 27^th^ March, national authorities declared a cholera outbreak in Beira City. As of April 21^st^, 6,768 cholera suspected cases (attack rate: 571 per 100,000 population) and 8 deaths (case fatality: 0.12%) had been reported in four districts of Sofala [3]. On May 1^st^, a cholera outbreak was declared in Cabo Delgado Province. As of July 14^th^, 284 cases were reported, with no deaths [4]. Over the years the country has been the target of natural disasters. In 2013, catastrophic flooding affected Gaza Province, resulting in the displacement of an estimated 120,000 inhabitants. On January 2015, the country was also seriously affected by Cyclone Chedza with a wind speed of around 90 Kmph, caused heavy rainfalls resulting in 140 deaths and left vast infrastructural damage [5]. Those recurrent events demonstrate that the country’s vulnerability to natural disasters and underlines the need for a functional early warning system and health system preparedness.

As part of an effort to strengthen the national respond to public health emergencies and communicable disease outbreaks, the Mozambican Ministry of Health (MoH), through the National Institute of Health (NIH) implemented a Field Epidemiology and Laboratory Training Program (FELTP). Beginning in 2010, the FELTP sought to build capacity in public health surveillance, epidemiology, disease control, and response to outbreaks and public health emergencies through training in applied epidemiology and laboratory management [6]. Worldwide, FELTPs are recognized as having technical expertise to respond to outbreaks and public health emergencies through timely risk assessments, rapid interventions, and development and use of outbreak specific field tools [7]. Effective preparedness for and response to infectious disease outbreaks require a public health workforce well trained in the principles and practice of field epidemiology [7]. The presence of adequate numbers of skilled health care workers with the ability to serve as first responders in an emergency is a key determinant of a successful emergency response [8].

**The FELTP response to the cyclones:** the NIH responded rapidly to the emergency, deploying six FELTP graduates to the affected area within 48 hours after Cyclone Idai made landfall to conduct risk assessments, initiate surveillance for communicable diseases, and to investigate and control suspected outbreaks. Similarly, after Cyclone Kenneth, two graduates were deployed to Pemba City in Cabo Delgado Province on April 29^th^ to rapidly establish active surveillance for possible emerging infectious diseases. A rotation system was established whereby each epidemiologist spent two weeks in the field in the red zones. The rotation of FELTP teams provided freshly trained healthcare workers to work actively in the field in affected areas and maintain the physical and mental health of the team members. However, because those two occurs almost simultaneously, it overwhelms the local capacity epidemiological capacity to respond. So far, the Moz-FELTP program graduated 46 epidemiologists but not all were available, and those that were available had to be divided into two provinces and several districts. This number demonstrated to be insufficient, considering the magnitude and impact of the event. The initial situation was catastrophic in the areas most affected by Cyclone Idai, there was no phone communications, no water supply, and 80% of the electrical infrastructure was damaged, road connections were also cut off and there was no fuel available. During the first days, there was no communication between the central level team and the field team. The lack of a backup communication system for warning and emergency operations demonstrate a weakness in preparedness and challenge emergency coordination, response effectiveness monitoring of activities conducted by FELTP. For example, in Buzi District, Sofala Province road access was interrupted, and epidemiologists depended on the availability of helicopters to reach the affected areas. Pemba City was accessible via air, sea and road, but the access to some affected areas was particularly difficult (for example Ibo and Macomia). It was a challenge for the FELTP team to optimally prepare for outbreaks. It requires specialized training and an operational mindset focused on the development of formal procedures and guidelines.

**Rapid epidemiological assessment of displaced population health status:** the process of conducting risk assessments and prioritization of human resources and health measures is critical for effective management of health emergencies [9]. Initially, in Sofala Province, the FELTP supported the triage of the displaced population rescued by boats in the flooded regions. The objective was to conduct a rapid assessment of acute problems, observe for possible signs of outbreaks, track patients who needed treatment into care and guarantee the supply of the medicines in the accommodation centers. The assessment also supported the early identification of patients on HIV antiretroviral and tuberculosis treatment to ensure continued access to medication.

**Implantation of emergency epidemiological surveillance:** the NIH led the implementation of emergency epidemiological surveillance to quickly detect adverse health events in the four critical affected districts of Sofala Province: Beira, Buzi, Dondo and Nhamatanda in collaboration with WHO, Centers for Disease Control (CDC), “Médecins Sans Frontières (MSF)” and other multilateral partners. Four priority diseases were identified for enhanced surveillance and early detection: fever, diarrhea, cholera and malaria. Initially disease surveillance data were collected and analyzed by the epidemiologist using a paper-based system then transitioned to the *Early Warning Alert and Response System* (EWARS/WHO) and integrated with the m-alert (electronic national surveillance system), where the data was collected through smartphones and transmitted centrally for analysis. The EWARS system triggered an alert when a priority disease was detected and as soon as the alert was verified, the FELTP graduates established daily risk assessments at the health facility, and verified, monitored, discarded or closed the alerts produced by the EWARS system. Daily and weekly epidemiological bulletins as well weekly Situational Report (SITREP) were produced using the compiled and analyzed data from the system.

**Data analysis to inform interventions:** an example of the utility of this system was demonstrated after the first cholera outbreak was declaration in Sofala Province. The FELTP team monitored and analyzed surveillance data and implemented the international standard cholera outbreak investigation protocol, investigated rumors, performed contact tracing, visited specific communities to conduct verbal autopsy and conducted environmental surveillance. This information was used to guide control measures taken by the Water, Sanitation and Hygiene (WASH) team and provided epidemiological information for the vaccination campaign against cholera. Similar activities, including the implementation of EWARS and m-alert, outbreak investigations, coordination with the WASH teams were conducted in Pemba City in the response to Cyclone Kenneth.

**Development of new tools:** although cholera was the main disease under surveillance, an increase in the number of bloody diarrhea cases was observed in Pemba district. A case definition was developed and implemented to monitor for typhoid fever. FELTP investigated cases of possible typhoid fever in Pemba district health facilities, developed a new investigation form to report, conducted investigations, confirm the cases and verify if it was an outbreak. Epidemiological intelligence and strategic planning of FELTP made it possible to act with accuracy and precision, as necessary. Examples included the development of an investigation form for bloody diarrhea and support of the laboratory team in establishing sentinel surveillance for acute jaundice in Sofala Province during the emergency.

**Conclusion:** in the context of the response to the cyclones, the Moz-FELTP has proven to be an important strategy to develop a locally trained public health workforce for public health emergency response. Although there is an insufficient number of graduates, the Moz-FELTP team demonstrated leadership and strong commitment to their work during this response, which can be attributed to the competencies acquired during training and their ability to serve as emergency responders. The strength of the current FELTP graduates underscores the need to produce a critical mass of competent field epidemiologists at all levels of the health sector to respond to public health emergencies. It is also important to continuous strengthen the capacity in public health disaster risk preparedness, mitigation and post-emergency health system recovery. A more comprehensive FELTP program curriculum including training in incident management system, humanitarian emergencies and preparedness for international emergencies is likely needed to respond to emergent public health challenges. We strongly suggest that health partners support this investment in health workforce training to ensure a resilient health system that can effectively prepare for, respond to, and recover from health emergencies.
